# Are Rural Primary Care Providers Able to Competently Manage Common Illnesses? A Cross-Sectional Study in Rural Sichuan, Western China

**DOI:** 10.3390/healthcare10091750

**Published:** 2022-09-12

**Authors:** Yian Fang, Shaohua Jiang, Pei Jiang, Huan Zhou, Min Yang

**Affiliations:** 1 West China School of Public Health and West China Fourth Hospital, Sichuan University, Chengdu 610041, China; 2 School of Public Health, Peking University, Beijing 100191, China; 3 The First Affiliated Hospital, Xinjiang Medical University, Urumqi 833054, China; 4 School of Public Health, North Sichuan Medical College, Nanchong 637100, China; 5 Faculty of Health, Design and Art, Swinburne University of Technology, Melbourne 3122, Australia

**Keywords:** primary care providers, competency, rural China

## Abstract

Background: Strengthening primary care is a key focus of the latest healthcare reforms in China. However, many challenges, including the workforce competence, still exist. This study aimed to evaluate the common disease management competency of rural primary care providers in Sichuan Province, western China. Methods: A cross-sectional study was conducted in 9 township health centers and 86 village clinics in 3 counties. Diarrhea and respiratory infection were selected as the evaluation cases. General partitioners were assessed through their abilities to (1) take history; (2) make diagnoses; (3) propose treatment; and (4) deal with clinical cases. Results: In total, 362 healthcare workers were surveyed, and 130 general practitioners were enrolled into our study. On average, rural primary care providers could only answer 46.4% of questions absolutely correctly, with 29.7% partly correctly and 23.8% incorrectly. Conclusion: We suggest strengthening training to improve rural primary care providers’ competencies, especially their capacities of history taking. Policy action is also needed to address regional disparities.

## 1. Introduction

Having been recognized as a core component of health systems since the early part of the twentieth century, primary care is an effective means to balance the two goals of the health service system—optimization of health and equity in distributing resources [1]. Even though it is taken as a main driver for universal health coverage, the development of primary care is uneven between and within countries.

Strengthening primary care is a key focus of the latest healthcare reforms in China [2,3]. During the COVID-19 pandemic, the importance of a people-centered integrated care system became much more apparent [4]. Since the founding of the People’s Republic of China in 1949, the delivery of healthcare has been organized around a three-tier public healthcare system, with a focus on primary care [5]. In rural areas, the major primary care providers (PCPs) are general practitioners (GPs) in village clinics (VCs) and township health centers (THCs), while county hospitals provide specialist medical services. THCs, as the middle cornerstone, offer comprehensive clinical care for common diseases, and provide technical guidance and supervision to VCs. VCs, as the bottom level, facilitate the primary care for frequent diseases such as the common cold, influenza, and diarrhea, etc. Both THCs and VCs are also responsible for public health services, contributing much to health promotion in rural areas [6]. Even though China does not have a compulsory gatekeeping regulation, statistics showed that only 2.7% of rural patients bypass the rural healthcare system [7]. Considering PCPs serve as the first point of contact for rural patients, it is of great importance to build a strong and robust primary care system. Although China has undoubtedly made remarkable progress in strengthening its primary care system [8], which also played key roles in the fight against COVID-19 [9], it still faces challenges in many aspects, including the quality of care [10].

It is widely accepted that, however measured, the quality of care in China could be enhanced [11]. Due to a range of historical and economic factors, health system development in rural areas has lagged behind that of urban areas [2]. PCPs in rural China are usually barefoot doctors who only received a little medical training [12], who may not be able to deliver high quality care. However, little evidence exists [3]. Recent years have seen a slow growth of studies exploring this topic. As the gold standard for assessing clinical practice [13], the method of unannounced standardized patients (USP) was adopted and low-quality healthcare offered by grassroots providers in rural China was illustrated [14]. Although this method has many advantages, policymakers cannot determine whether poor quality care is caused by low levels of technical knowledge and competencies or other factors such as satisfaction and incentives, leading to the difficulties in evidence-informed policy making.

As a key component of quality, workforce competency, however, was neglected in the previous literature [15]. PCPs competencies are usually evaluated through vignettes, hypothetical cases providing brief description of symptoms [15], which has proven accurate and inexpensive [16]. There are two approaches to using vignettes: one is through interaction between PCPs and simulated patients, while the other presents the vignettes directly to PCPs. Even though the former can better simulate the real conditions, the latter is easier to conduct in low-income settings, requiring less resources. Many research studies have used vignettes to evaluate PCPs’ competency in other developing countries [17,18], but this field is still under-researched in China. The only four studies using vignettes demonstrated poor competencies of village clinicians [19,20,21,22], but did not evaluate GPs in THCs, which may not represent the overall situation of PCPs in China.

Therefore, our study aims to address PCPs’ competencies in detail, which are taken as the pre-requisite for implementing best practice and providing high-quality care [23]. Through stressing the details of competencies such as the ability to take histories, make diagnoses and provide treatments, we aim to provide guidance for future interventions.

This study is one of the first studies aimed at understanding different aspects of primary care competencies in rural China. Even though there are a growing number of studies in the literature focusing on China’s rural primary care quality, evidence to guide targeted interventions is still lacking. Previous studies fail to understand in depth the shortcomings of general practitioners’ competencies. This study analyzes the competency by focusing on taking histories, making diagnoses, proposing treatments and managing clinical cases, which contributes to the literature of quality measurement. We aim to provide clear guidance on how to design interventions to improve the competencies of PCPs in rural China.

## 2. Methods

### 2.1. Study Setting and Participants

We select Sichuan as an example to assess the situation in western China. Sichuan is a typical agricultural province with a large population in the western region of China, which is thought to be a scaled-down model of the entire country in many aspects [24]. The total area of Sichuan Province is 486 thousand square kilometers with a population of 83.7 million in 2020. There are major disparities in the levels of economic development and the availability of healthcare resources throughout this province [25], which can be roughly divided into two geographic/demographic zones. West Sichuan is a vast, rural, mountainous, sparsely populated, high-altitude, ethnic minority region with a less-developed economy and healthcare system. While east Sichuan, the non-minority region, is a densely populated plain with well-developed economy and healthcare system. In 2016, Sichuan had 4490 township health centers and 55,960 village clinics with 195 million visits in rural primary care centers [26]. It is of great importance to investigate the primary care quality in Sichuan and to inform the development and reform of the rural health service system in western China.

Purposive sampling was used to determine study sites. At the first stage, terrain and economic statuses were considered when choosing the three counties. Afterwards, taking accessibility and population characteristics into account, three towns within each county, stratified by distance to the county center were selected, which were Xihe, Huangtu and Baihe in Longquanyi County, a top level economic development district in east Sichuan (Zone A); Zhaohua, Gufo and Huzhu in Fushun County, a mid-level economic development agricultural county (Zone B); and Xinshiba, Qianjin and Sijue in Ganluo County, a poor ethnic county in the mountain area of west Sichuan (Zone C) (Table S1 and Figure S1). The main THC and all VCs in sample towns were included. In total, 362 healthcare workers in 9 township health centers and 86 village clinics joined this study. Admittedly, our study may not be a perfect representation of rural Sichuan due to the sampling method. This study, however, aims to compare the PCPs’ competencies among different terrains and economic development statuses and examine the inequity issues to inform policymaking.

To enable comparison between different facilities, we tried to evaluate the competencies of PCPs through standardized cases. Considering almost all rural PCPs in China are trained as physicians, internal medicine cases were chosen, and surgeons, nurses and other healthcare workers were excluded. Because clinical guidelines issued by the government were mainly based on western medicine, traditional Chinese or ethnic medicine doctors were not enrolled into our study. With the support of heads of THCs, all GPs in THCs and VCs were included in our study, amounting to 130 in total. Electronic questionnaires were sent to all participants via smart phone, which have been validated by our colleagues [27]. Those who were not able to access the internet were supplemented with paper questionnaires. Two trained researchers (M.Y. and a graduate) were on site during the period of data collection to help with operational questions when asked by participants. The survey period was from March to June 2017.

### 2.2. Cases

Diarrhea and respiratory infection were selected to match previous studies conducted by Sylvia et al. [14] and Currie et al. [28]. In the study by Sylvia and colleagues, they trained individuals to serve as incognito patients presenting with symptoms of dysentery and angina and found that the quality of care was low. Currie and colleagues conducted an audit study in which a pair of simulated patients with identical influenza-like complaints visited the same general practitioner and found that a patient who displayed knowledge of appropriate antibiotics use reduced both antibiotic prescription rates and drug expenditures. To find out whether these results were due to the low competency of GPs, these two symptoms—diarrhea and respiratory infection—were chosen. It is also worth noting that diseases with diarrhea (such as dysentery) and respiratory infection (such as influenza) are highly relevant to the current and future disease burden in rural China. If PCPs cannot manage these common illnesses, it may indicate their overall technical competencies are to some extent unsatisfactory.

### 2.3. Measures

In order to evaluate the technical competencies, PCPs were assessed for their ability to (1) take history; (2) propose differential diagnoses; (3) treat a patient adequately; and (4) deal with clinical cases (Table 1). These answers were in the text format and were translated into quantitative data for further analyses. Based on the clinical guideline issued by the government [29,30,31], a checklist with essential items was designed to judge whether care providers’ answers were “absolutely correct”, “partly correct” or “incorrect”. Cases and checklists were selected from authorized internal medicine textbooks [32] and their matched workbooks by two senior clinical medicine students, and were validated under the supervision of clinical experts. For the first three parts—history taking, differential diagnoses and treatment schemes—covering all essential items in the checklist would be taken as “absolutely correct”; only covering some essential items or mentioning items not recommended would be taken as “partly correct”; covering none or mentioning wrong items would be taken as “incorrect”. For cases of diagnoses and treatments, if the GP mentioned the main disease and correct treatments, it would be taken as “absolutely correct”, and “partly correct” for similar diseases or treatments. For example, dyspepsia would be taken as “partly correct” if what the patient suffers from is physiological diarrhea (both are non-infectious). However, dysentery would be a wrong diagnosis considering it is infectious. Competency scores were calculated through the following process: (1) weighting absolutely correct as 2, partly correct as 1, while incorrect as 0; and (2) add all scores, knowledge scores, and case scores as three indicators. If the PCP answered all questions absolutely correctly, the score would be 28, while the minimum score would be 0 if all answers were incorrect. Data abstraction and evaluation was conducted by two researchers (P.J. and S.J.) independently. Discrepancies of 4.1% from double entry were found, which were corrected by Y.F. based on the checklists. Consistency was guaranteed by reproducibility. The participants were also asked for background information including date of birth, sex, education, professional title, certificate, full time/part time, income status and work time. We took Sichuan Health and Family Planning Statistical Yearbook 2016 as references to determine the categories of these variables.

### 2.4. Statistics Analyses

Considering sample counties can to some extent represent three varieties with different economic development zones—rich, median and poor—the comparison quality of care in these regions was conducted. Meanwhile, even though village clinics and township health centers play different roles in China’s rural healthcare system, their basic functions were similar—to make diagnoses and provide basic treatments. In this case, we also compared the characteristics and competency of PCPs in these two types of facilities. Univariate statistics of all variables grouped by region and varieties of health facilities were conducted separately, using Fisher exact test to compare proportions and Kruskal–Wallis H test or Mann–Whitney U test to compare median numbers (α = 0.05). The descriptive statistics of competency questions were also presented. We also conducted ordinal least regressions to identify potential influencing factors of the competency. All analyses in this study were conducted using STATA 15.1 (StataCorp LLC., College Station, TX, USA).

## 3. Results

### 3.1. Primary Care Providers’ Characteristics

The differences of all variables by VCs and THCs are presented in Table 2. The education statuses for GPs in THCs are better, with 33.9% holding degrees of bachelor or above, 37.5% junior college and 28.6% technical secondary school or below. In comparison, most village clinicians obtain a degree from technical secondary school or below and only 1.4% hold bachelor’s degree or above. As for professional title, no GPs surveyed have senior titles. None of the 16 practitioners who have intermediate titles work in VCs, and 64.3% GPs in THCs have a primary title while the figure is 55.4% in VCs. Moreover, 44.6% of village doctors and 7.1% of town doctors do not have any technical titles. As for income status, GPs in THCs receive more salary than their peers on average, but the workloads of village physicians are greater.

### 3.2. Primary Care Providers’ Competency

On average, rural GPs in this study could only answer 46.4% (6.5 out of 14) questions absolutely correctly, with 29.7% partly correctly and 23.8% incorrectly. Details of the general practitioners’ adherence to clinical guidelines can be found in Figures S2–S15. When we further divided the questions into two parts: knowledge (six questions including history taking, differential diagnoses and treatment schemes); cases diagnoses and treatments (eight questions), we find that the likelihood of answering incorrectly for knowledge questions is 39.4%, while the percentage is 12.3% for case management questions.

To facilitate comparisons, competency scores were calculated. Table 3 presents scores in different regions. Generally, PCPs in Zone C, the relatively undeveloped region, performed worse than the other two regions. However, Zone A and Zone B had better economic situations, and even their scores are only half of the maximum score.

The competency comparisons between VCs and THCs are presented in Table 4. Though most practitioners surveyed have certificates for medical services, among 14 questions, PCPs in VCs and THCs can only answer 44.2% and 49.2% questions absolutely correctly, 33.4% and 24.9% partly correct, while 22.2% and 25.9% incorrectly, respectively. Specifically, GPs in VCs performed slightly better in treatment knowledge, but worse for differentiate diagnoses. VCs and THCs did not show significant differences in competencies for clinical cases diagnoses and treatment.

The influencing factors of PCPs competency is presented in Table S2. However, due to the relatively small sample size, we did not find significant results.

## 4. Discussions

Primary care is the key to achieving universal health coverage, and the high-quality workforce is the foundation. Our results show the poor common disease management competencies of PCPs in the three counties of Sichuan. Considering these two symptoms are common, the result indicated the overall services they deliver may not be satisfying, which has also been shown by previous studies [19,20,21,22]. One surprising result is that even though GPs are equipped with low-level medical knowledge, they are still able to correctly manage case vignettes in terms of diagnoses and treatment, considering their performances in these two scales are relatively acceptable (12.3% incorrect rate). However, it is worth noting that the questionnaire includes all the information (e.g., history, symptoms) needed to diagnose and treat. Considering only 32.3% of PCPs can give absolutely correct answers for history taking, when encountering a real patient, they may not be able to make the correct diagnoses and treatments as shown in the study, as patients may not be able to offer the necessary information by themselves in the actual practice. This finding is consistent with previous studies, indicating the poor quality of diagnosis process [22]. Therefore, targeted training on history taking abilities should be organized in primary care settings. Research has found that simple dissemination of written guidelines would be ineffective, and supervision and audit with feedback is preferred [33]. As used in previous studies [34], China can implement tailored continuing training through online platforms to improve the skills of healthcare workers, with feedback mechanisms. As the telemedicine-based diagnoses assisting system has proved effective in other settings [35], China can also consider the wide adoption of the technology to provide high-quality care to rural populations. A performance accountability system can be established to monitor and incentivize competence improvements.

The disparities in rural GPs’ competencies found in the study are mainly between ethnic and non-ethnic regions. Similar variations in competency were also found in previous studies [15]. China is a multi-ethnic country with 56 ethnicities. Many efforts have been made to reduce the ethnic disparities in the quality of healthcare in China; however, inequities in healthcare provision continue to exist. With a huge disparity in spatial accessibility to care between ethnic minority and non-minority regions in China [25], more attention is needed in ethnic minority areas.

The study does not present significant differences in GPs’ competencies between village clinics and township health centers, though both do not perform well. This may be for two reasons. On the one hand, the cases selected are common diseases in rural China, so there would not be significant differences between PCPs in THCs and VCs. On the other hand, it may be because in rural China, township PCPS provide frequent training, often monthly, for village PCPs. This approach has been highlighted in many integrated healthcare systems in China, which is also the future direction of China’s primary care reform [11]. In resource constraint settings, the approach of arranging intensive training to township doctors and providing incentives for them to train village doctors can be a cost-effective way to improve the competencies of rural care providers.

This study finds a low education level of PCPs in rural Sichuan, widely cited as the key reasons for poor competency [11]. PCPs in VCs have limited formal medical education, thus lack the ability to diagnose and treat appropriately. Meanwhile, even though most PCPs in VCs have professional qualifications, 20% of providers are still unqualified. Past research has summarized two interventions to improve health worker knowledge and skills, including enhanced training and selective licensing [33]. Our study indicates the necessity of both approaches. The situation in THCs is better. Almost half of PCPs in THCs receive bachelor’s degrees or higher, under the efforts of governments. The government also provides some continuing training opportunities for PCPs to improve their abilities, which can contribute to competency enhancement in rural areas.

Meanwhile, similarly to Zhu et al. [36], the study finds a heavy workload of rural PCPs, especially those in village clinics. However, their income to some extent cannot match their dedication. Although the governments have increased the subsidies to rural providers, the compensation is still relatively low in contrast to the heavy workload [12]. Furthermore, pensions are not widely provided to PCPs. This may be the main reason for widespread low satisfaction and high burnout among PCPs [37]. Even though China has issued some policies to attract young medical graduates to practice in rural areas such as waiving their tuition [3], the impacts would be small if PCPs are not able to have corresponding salary and career development opportunities. Future policies should also take these challenges into account.

Our study also has some limitations. First, the study sample is not representative of rural western China. However, by taking terrains, economic statuses, and population characteristics into account, it reflects the status of rural Sichuan Province, western China, well. Secondly, to match the diseases adopted by previous studies, we only select diarrhea and respiratory infection to evaluate the competencies of general practitioners. Although they are common in rural China, it cannot comprehensively reflect primary care providers’ competencies. Thirdly, considering the clinical guidelines issued by the government are mainly based on Western medicine, our study excludes traditional medicine doctors, though around 15% of clinicians in western China specialize in traditional Chinese medicine (7.7% nationally) [13]. Future studies should select more varieties of diseases and draw larger samples in a wider region to obtain more precise data in relation to primary care providers’ competencies in rural China.

## 5. Conclusions

Our study illustrates that rural primary care providers in rural Sichuan may not be prepared to act as the front-line fighters against common diseases in the three-tier healthcare system. Although their case management knowledge is relatively acceptable when all information is provided, patients in actual practice do not know what is necessary for general practitioners to diagnose and treat accurately, and usually fail to provide all the necessary information. Therefore, future training should focus more on building the capacity in history taking. The heavy workload and low salary of rural primary care providers also need more attention.

## Figures and Tables

**Table 1:** Questions to measure technical competency.

Competency	Diarrhea	Respiratory Infection
History taking	What will you ask encountering a patient complaining with diarrhea?	What will you ask encountering a patient complaining with respiratory infection?
Differential diagnoses	How to distinguish infectious and non-infectious diarrhea?	How to distinguish influenza and common respiratory infection?
Treatment	How to treat acute diarrhea (mild dehydration)	How to treat influenza?
Cases diagnoses and treatments (1) What disease did the patient suffer from? (2) How would you treat this patient under this circumstance?	Baby boy, 3 months old, had a weight of 5.6 kg. Mixed feeding was adopted. Having diarrhea for 2 months, he defecated 5 to 6 times per day. Loose or sticky stools without pus and blood were found. He had a good appetite and had eczema on his face.	Female, 24 years old. She started to have a mild sore throat yesterday morning and began to have a temperature over 37 °C at that night. This morning, the pain relieved to some extents. Her symptoms included a slight fever, runny nose and cough. A mild red throat was found but cardiopulmonary auscultation was normal. Lymphoid follicles were visible at posterior pharyngeal. Cervical lymphadenopathy was not found.
	Baby boy, 3 months old. Bottle feeding was adopted. Two days ago, his stool frequency increased. Loose stools with milk cured were found. He had a stomach-ache with a bad appetite. He always cried and screamed without a sound sleep. Sometimes the baby vomited. No fever or dehydration was found. The microscopic examination of stools showed fat granules and a small amount of leukocytes.	Male, 34 years old. Five days ago, he began to cough and had a fever with a running nose and a sore throat. Two days later, the conditions eased but his nose was still running. This morning, his left facial began to ache, and the symptom of nose running deteriorated with a temperature of 38 °C. His symptoms included cough with expectoration, phlegm adhering to the pharynx and a running left nose, but his throat was not sore. A mild red throat was found and lymphoid follicles were visible at the posterior pharyngeal, with left maxillary sinus tenderness (+), but cervical lymph nodes cannot be touched.

Notes: PCPs are asked to answer these questions using the best of their knowledge, regardless of the rural settings.

**Table 2:** Univariate analyses grouped by township health centers and village clinics.

Variables	VCs N (%)	THCs N (%)	*p* Value (Fisher Exact Text)
County			0.033
Zone A	25 (33.3)	22 (39.3)	
Zone B	50 (66.7)	30 (53.6)	
Zone C	0 (0.0)	4 (7.1)	
Gender			0.072
Male	50 (66.7)	28 (50.0)	
Female	25 (33.3)	28 (50.0)	
Age			<0.001
Under 35	1 (1.3)	20 (35.7)	
35–44	40 (53.3)	23 (41.1)	
45–54	26 (34.7)	12 (21.4)	
Above 55	8 (10.7)	1 (1.8)	
Education *			<0.001
Technical secondary school or below	64 (85.3)	16 (28.6)	
Junior college	8 (10.7)	14 (25.0)	
Bachelor or above	3 (4.0)	26 (46.4)	
Professional title			<0.001
Intermediate	0 (0.0)	16 (28.6)	
Primary	42 (56.0)	36 (64.3)	
No title	33 (44.0)	4 (7.1)	
Professional qualification			0.057
Have	62 (82.7)	53 (94.6)	
Do not have	13 (17.3)	3 (5.4)	
Years since qualification issued			<0.001
Under 5	20 (26.7)	14 (25.0)	
5–9	2 (2.6)	18 (32.1)	
10–19	23 (30.7)	17 (30.4)	
20 or above	30 (40.0)	7 (12.5)	
Work status			0.034
Full time	61 (81.3)	53 (94.6)	
Part time	14 (18.7)	3 (5.4)	
Income status			<0.001
Under RMB 1500 (USD 236.9)	20 (26.7)	0 (0.0)	
RMB 1500–2499 (USD 236.9–394.6)	21 (28.0)	3 (5.4)	
RMB 2500–3499 (USD 394.7–552.5)	21 (28.0)	13 (23.2)	
RMB 3500–4499 (USD 552.6–710.4)	7 (9.3)	16 (28.6)	
RMB 4500 or above (USD 710.5)	6 (8.0)	24 (42.9)	
Workload in time			<0.001
≤ 8 h per day	16 (21.3)	39 (69.6)	
> 8 h per day	59 (78.7)	17 (30.4)	

* Notes: In China, there are three levels of formal medical training for primary healthcare doctors: medical college (5 years of medical education after 12 years of primary and secondary education to obtain a bachelor’s degree of medicine); junior medical college (3 years of medical education after 12 years of primary and secondary education); and technical school (3 years of medical education after 9 years of primary and secondary education). The specialty is usually family medicine.

**Table 3:** Comparison of competency scores among economic zones.

Competency Scores	Zone A Median (25–75%) (N = 47)	Zone B Median (25–75%) (N = 80)	Zone C Median (25–75%) (N = 4)	*p* Value (Kruskal–Wallis H Test)
Total (Max. 28)	18 (15–19)	17 (15–20)	12 (10–13.5)	0.017
Knowledge (Max. 12)	6 (5–8)	7 (5–8)	5 (4–6)	0.306
Taking history (Max. 4)	2 (1–3)	2 (1–3)	2 (1–3)	0.779
Differentiate diagnosis (Max. 4)	2 (2–4)	2 (2–4)	3 (2–4)	0.424
Treatment scheme (Max. 4)	2 (1–3)	2 (2–3)	0 (0–0)	0.003
Clinical cases (Max. 16)	11 (10–13)	10 (9–12)	6 (5–8.5)	0.006
Diagnoses (Max. 8)	6 (5–7)	5 (4–7)	4 (3.5–4.5)	0.033
Treatment (Max. 8)	6 (5–6)	5 (5–6)	2 (1.5–4)	0.036

**Table 4:** Comparison of competency between village clinics and township health centers.

Competency Scores	VCs Median (25–75%) (N = 75)	THCs Median (25–75%) (N = 56)	*p* Value (Mann–Whitney U Test)
Total (Max. 28)	17 (15–20)	17 (15–19)	0.844
Knowledge (Max. 12)	7 (5–8)	7 (5–8)	0.780
Taking history (Max. 4)	2 (1–3)	2 (1–3)	0.436
Differentiate diagnosis (Max. 4)	2 (0–2)	4 (2–4)	<0.001
Treatment scheme (Max. 4)	2 (2–4)	2 (2–2)	0.014
Clinical cases (Max. 16)	10 (9–12)	11 (9–13)	0.789
Diagnoses (Max. 8)	6 (5–7)	5 (4–7)	0.788
Treatment (Max. 8)	5 (5–6)	6 (4.5–6)	0.244

## Data Availability

Data are available under reasonable request to the corresponding author.

## Supplementary Materials

The following supporting information can be downloaded at: https://www.mdpi.com/article/10.3390/healthcare10091750/s1, Table S1: Characteristics of sample counties in 2014; Table S2: Regression results of competency scores; Figure S1: Study Sites in Sichuan Province; Figure S2: What will you ask encountering a patient complaining with diarrhea? Figure S3: How to distinguish infectious and non-infectious diarrhea? Figure S4: How to treat acute diarrhea (mild dehydration)? Figure S5: What will you ask encountering a patient complaining with respiratory infection? Figure S6: How to distinguish influenza and common respiratory infection? Figure S7: How to treat influenza? Figure S8: Diagnoses of Case 1; Figure S9: Treatments of Case 1; Figure S10: Diagnoses of Case 2; Figure S11: Treatments of Case 2; Figure S12: Diagnoses of Case 3; Figure S13: Treatments of Case 3; Figure S14: Diagnoses of Case 4; Figure S15: Treatments of Case 4
