# Supplementary material for: Are Rural Primary Care Providers Able to Competently Manage Common Illnesses? A Cross-Sectional Study in Rural Sichuan, Western China
Source: Healthcare (Basel). 2022 Sep 12;10(9):1750. doi: 10.3390/healthcare10091750 (PMC9498850; doi:10.3390/healthcare10091750)
Supplement: Supplementary file 1 [file healthcare-10-01750-s001.zip › healthcare-1852488-supplementary.pdf]

healthcare

MDPI

Table S1. Characteristics of sample counties in 2014

	Longquanyi County	Fushun County	Ganluo County
Terrain	Plateau	Hills	Mountains
Percentage of ethnicity (%)	0.9	0.1	76.7
Gross domestic product per capita (yuan)	117,854	24,928	12,781
Percentage of agricultural populations (%)	47.8	75.9	93.0
Permanent populations (10 thousand)	63	110	23
Numbers of hospitals	24	11	2
Numbers of township health centers	9	26	28
Numbers of village clinics	113	673	227
Populations served by THCs and VCs (10 thousand)	7.00	4.23	0.82

Date Source: Statistics Yearbook 2014 of Chengdu City, Zigong City and Liangshan Yi Autonomous Prefecture.

Figure S1. Study Sites in Sichuan Province

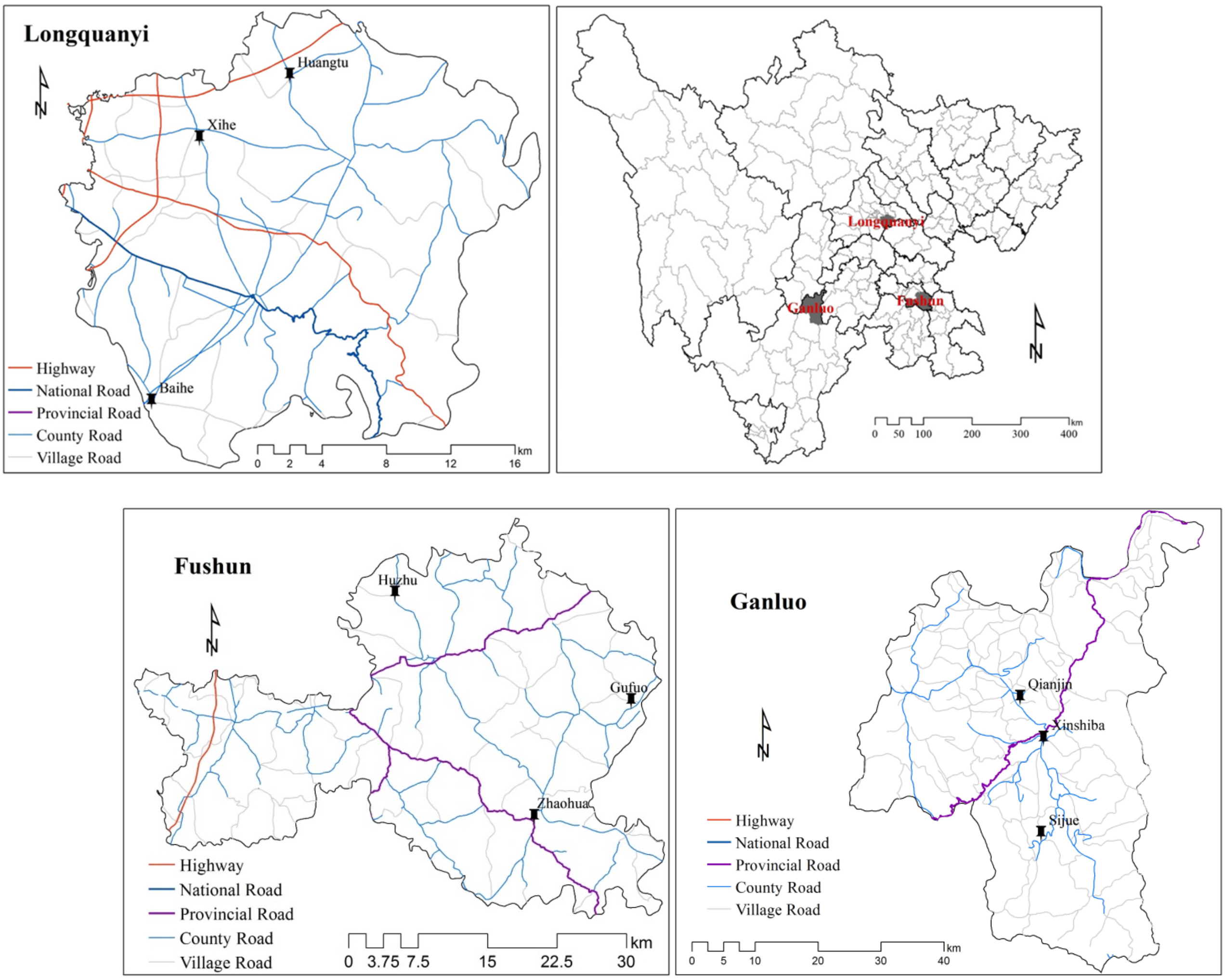

Table S2. Regression results of competency scores

	Sqrt (Total)<br>Coef. (SE)	Knowledge<br>Coef. (SE)	Clinical case<br>Coef. (SE)
THC (vs VC)	0. 054 (0.104)	0.225 (0.569)	0.181 (0.545)
Education year	0.001 (0.025)	-0.017 (0.134)	0.024 (0.128)
Professional title			
Primary (vs intermediate)	-0.181 (0.124)	-1.121 (0.678)	-0.398 (0.649)
No title (vs intermediate)	-0.061 (0.153)	-0.551 (0.837)	-0.016 (0.801)
Professional qualification (vs Yes)	-0.122 (0.118)	-0.271 (0.642)	-0.733 (0.615)
Zone Fixed effects	Yes	Yes	Yes
N	131	131	131
F statistics	2.55	0.96	2.75
R-squared	0.127	0.052	0.136

Specifically, Figure S2-S15 show the general practitioners' adherence to clinical guidelines. Some general answers are not included. For example, if a doctor only mentions "symptoms" but fails to explain what kind of symptoms the client has, this answer would be invalid.

General practitioners' answers to the question "what will you ask encountering a patient complaining with diarrhea" are presented in Figure S2. Stool frequency, stool type and fever are taken as essential items, but only 58.1% and 60.7%, 39.2% and 53.6%, 12.2% and 12.5% physicians in village clinics and township health centers mention these items respectively. As for "how to distinguish infectious and non-infectious diarrhea", merely 50.0% and 71.4% village and town primary care providers mention "examination", the essential item. Moreover, some GPs mention wrong items such as "abdominal pain" and "tenesmus" which cannot help differentiating these two kinds of diarrhea (Figure S3). Figure S4 illustrates the treatment for acute diarrhea (mild dehydration). Oral Rehydration Salt (ORS) is the most appropriate treatment and almost 90% doctors in VCs (85.1%) and THCs (89.3%) mention that. Some GPs mentioned "antibiotics" which are generally not recommended and some mentioned "antidiarrheal medicines" and "fasting", which are wrong.

As for respiratory infection, general practitioners' answers are presented in Figure S5-S8. Fever is considered as an essential item when diagnosing respiratory infection and 62.2% and 53.6% doctors in village clinics and township health centers refer to it (Figure S5). Figure S6 illustrates when practitioners are asked "how to distinguish influenza and common respiratory infection", what their answers are. The prevalence or infectiousness is taken essential, and 52.7% and 73.2% GPs in VCs and THCs refer to it. Considering most rural healthcare facilities do not have appropriate equipment to conduct examination, the varieties of viruses are not taken necessary. As for "how to treat influenza" (Figure S7), antivirus medicines are necessary, and patients should take as soon as possible if suffering from influenza. But only 62.2% and 30.4% village and town practitioners mention it.

Figure S8-S15 show the answers for case diagnoses and treatments. Case 1 is a baby suffering from physiological diarrhea. Only 23% village doctors and 37% town doctors diagnose correctly (Figure S8). Physiological diarrhea doesn't need specific treatment and clinical observation is enough. However, less than 10% GPs in VCs and THCs refer to it, which shows that among those who diagnosed this correctly, some failed to provide the appropriate treatment regimen (Figure S9). Case 2 presents a baby suffering from dyspepsia. 64% village care providers' diagnoses were correct, while only 45% town care providers knew the symptoms were caused by "dyspepsia" (Figure S10). Moreover, a large percentage of physicians (18% of VCs and 36% of THCs) take it as "infectious diarrhea", "bacterial diarrhea", "dysentery" or "enteritis", which is incorrect. For treatment, No more than 40% general practitioners mention "diet adjusting" and less than 10% refer to "probiotics" which are taken essential (Figure S11). What are worse, only 9.5% village doctors

and 25% of town doctors recommend the use of antibiotics for treatment, which may do great harm to babies suffering from the same disease. Case 3 and Case 4 present two patients suffering from respiratory infection. The first case is common cold with pharyngolaryngitis/tonsillitis while the second case is genyantritis/nasosinusitis. Antibiotics are necessary in Case 4 but not recommended in Case 3. However, though 89% village practitioners and 91% town practitioners can make totally or partly correct diagnoses (Figure S12), 12.2% and 23.3% would prescribe antiviral medicines and 13.5% and 7.1% antibiotics, which generally is not recommended (Figure S13). As for Case 4, no more than 40% GPs were able to mention the correct diagnosis (Figure S14) though 31.1% village doctors and 57.1% town doctors know antibiotics are the best way to treat these symptoms (Figure S15).

Figure S2. What will you ask encountering a patient complaining with diarrhea?

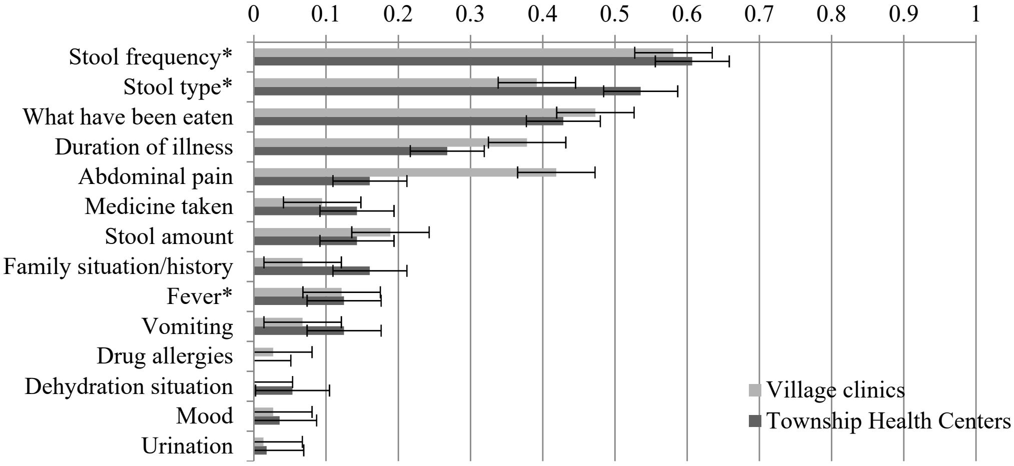

Notes: Figure shows means and 95% confidence intervals for clinician adherence to diarrhea case clinical checklist items. Items with asterisks (*) are considered essential. Six GPs mention tenesmus which is not noted in the guideline.

Figure S3. How to distinguish infectious and non-infectious diarrhea?

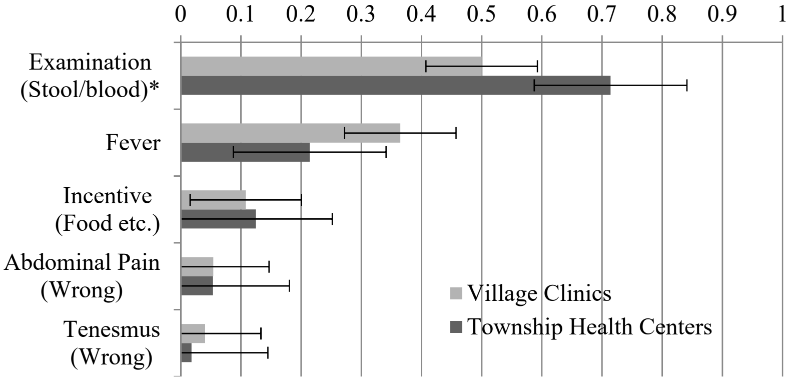

Notes: Figure shows means and 95% confidence intervals for clinician adherence to diarrhea case clinical checklist items. Items with asterisks (*) are considered essential. We cannot distinguish infectious and non-infectious diarrhea through abdominal pain and tenesmus.

Figure S4. How to treat acute diarrhea (mild dehydration)?

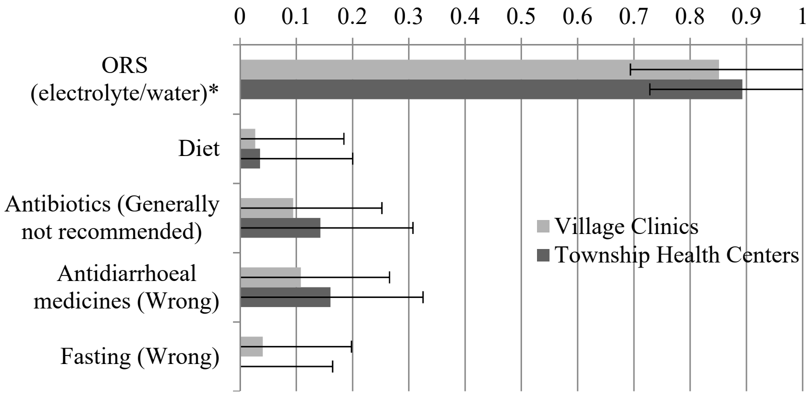

Notes: Figure shows means and 95% confidence intervals for clinician adherence to diarrhea case clinical checklist items. Items with asterisks (*) are considered essential. Antibiotics are only used when bacterial infection is confirmed and antidiarrheal medicines and fasting are wrong.

Figure S5. What will you ask encountering a patient complaining with respiratory infection?

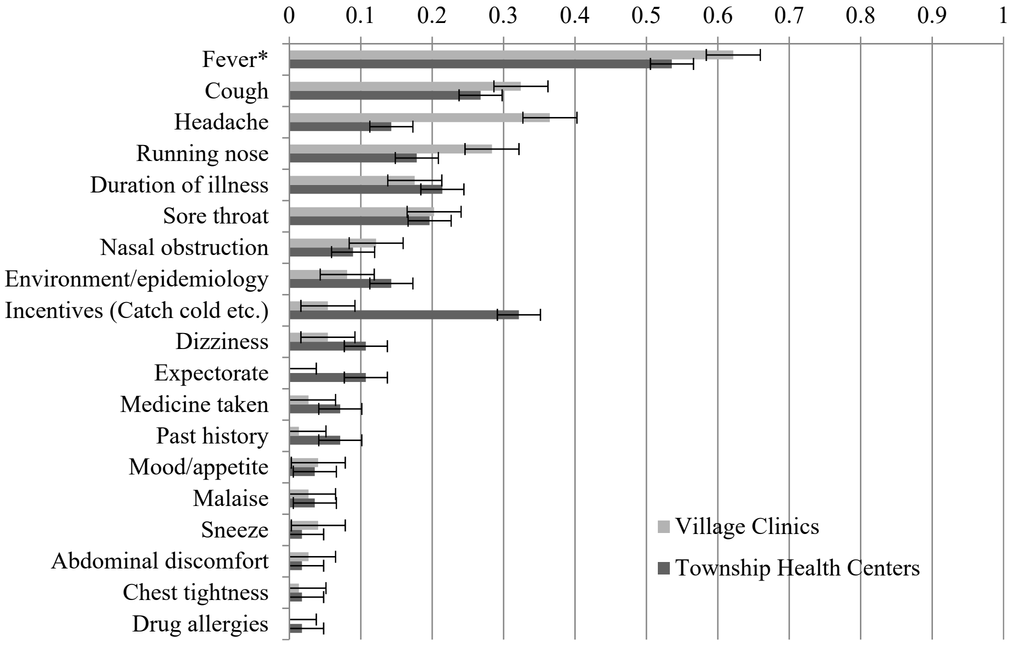

Notes: Figure shows means and 95% confidence intervals for clinician adherence to respiratory infection case clinical checklist items. Items with asterisks (*) are considered essential.

Figure S6. How to distinguish influenza and common respiratory infection?

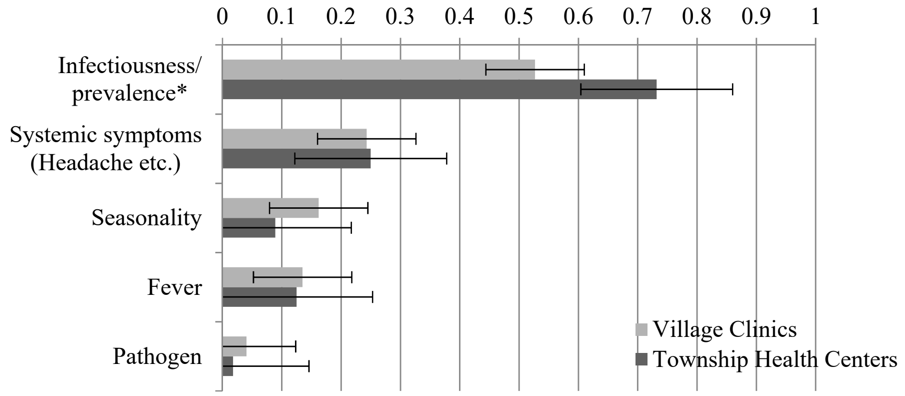

Notes: Figure shows means and 95% confidence intervals for clinician adherence to respiratory infection case clinical checklist items. Items with asterisks (*) are considered essential. Though 10 GPs mention the pathogens of common respiratory infection and influenza are different, 3 GPs think virus may only cause influenza but not common respiratory infection (not mention what cause common respiratory infection), and 1 GP thinks influenza is caused by virus, and common respiratory infection is caused by bacteria. Influenza and common respiratory infection are caused by different kinds of virus. What's more, the other two GPs only mention "pathogen" and we cannot distinguish whether they have the right answers. So, these answers are not included. Also, some GPs mention "duration of illness" which is not noted in the guideline.

Figure S7. How to treat influenza?

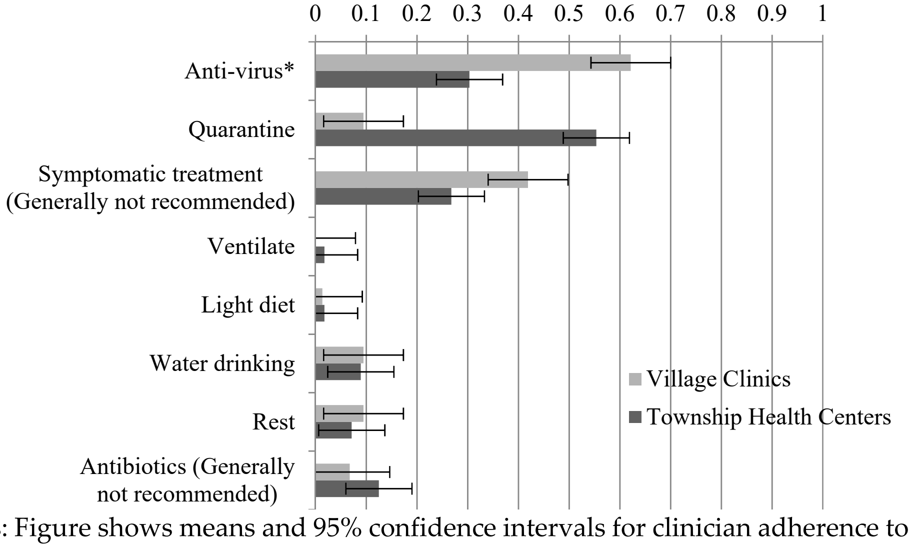

Notes: Figure shows means and 95% confidence intervals for clinician adherence to influenza case clinical checklist items. Items with asterisks (*) are considered essential. We used the 2011 version of guideline considering the study conducted in 2017 when the 2018 version is not adopted. In the guideline, symptomatic treatment is not recommended generally because the anti-virus medicines are usually specific.

Figure S8. Diagnoses of Case 1

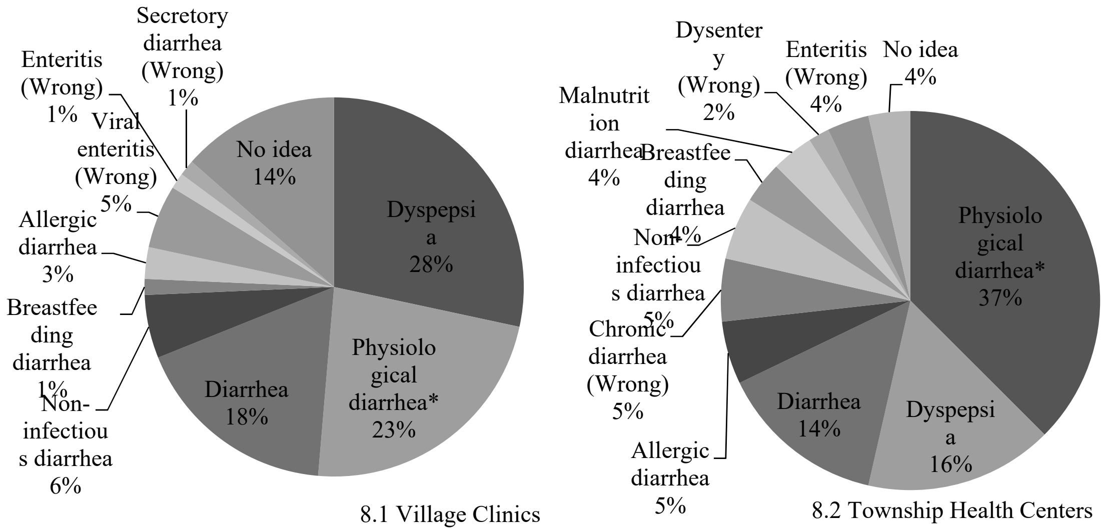

Notes: Case 1 is a baby suffering from physiological diarrhea (*). Some GPs only mention "diarrhea" and we also includes them. Dysentery, (viral) enteritis and secretory diarrhea are taken as wrong diagnoses.

Figure S9. Treatments of Case 1

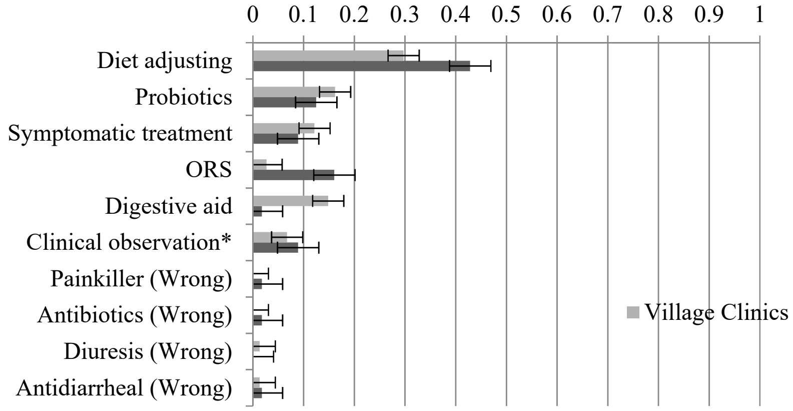

Notes: Considering Case 1 is a baby suffering from physiological diarrhea, clinical observation is the best way. In other words, no measures need to be taken. Actually adjusting diet, probiotics and something like that will not do harm to the baby, so we don't think them wrong. However, painkiller, antibiotics, diuresis and antidiarrheal may impair babies' health, so they are taken wrong.

Figure S10. Diagnoses of Case 2

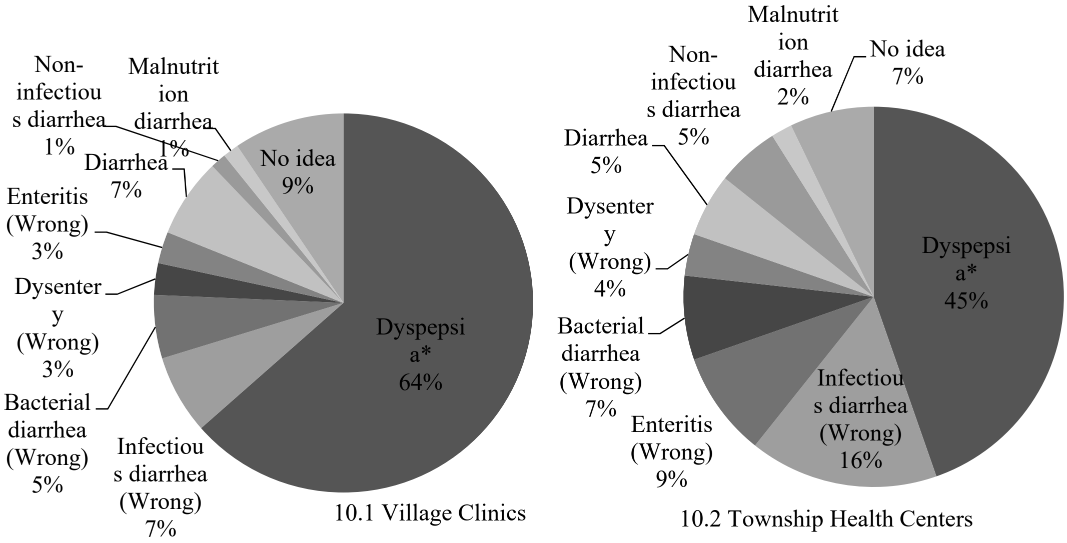

Notes: Case 2 is a baby suffering from dyspepsia (*). Some GPs only mention "diarrhea" and we also includes them. Infectious diarrhea, enteritis, bacterial diarrhea and dysentery are taken as wrong diagnoses.

Figure S11. Treatments of Case 2

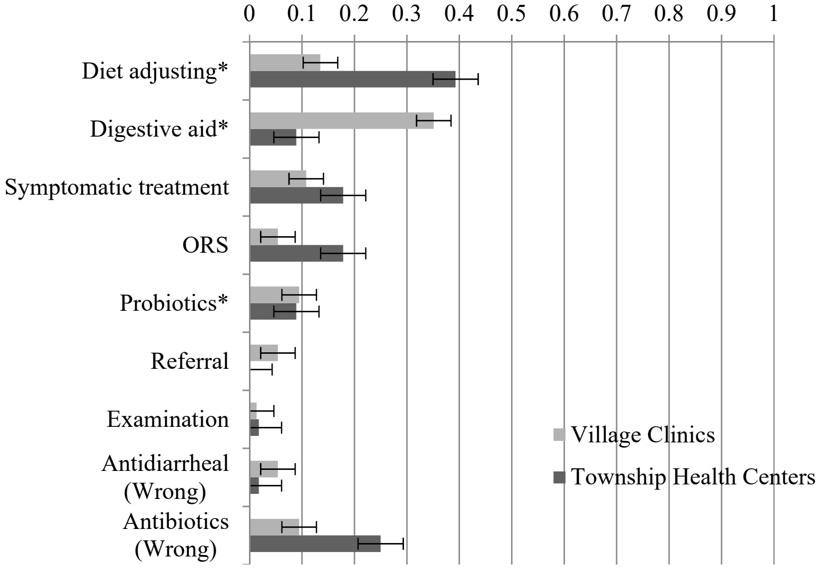

Notes: Considering Case 2 is a baby suffering from dyspepsia, diet adjusting is the best way. Adding probiotics is also recommended. ORS may not do harm to the baby, so we don't take them wrong. Referral and examination are also correct measures rural GPs can take. However, antibiotics, diuresis and antidiarrheal may impair babies' health, so they are taken wrong.

Figure S12. Diagnoses of Case 3

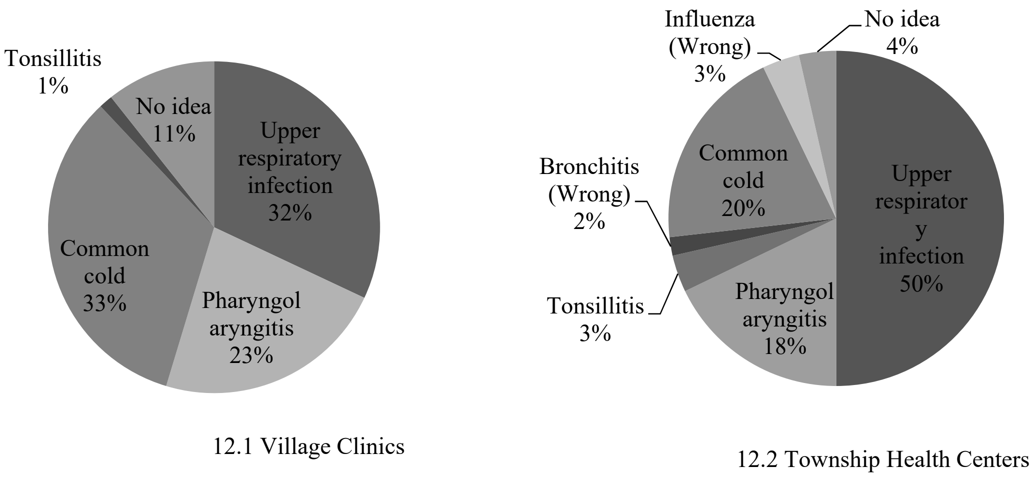

Notes: Case 3 is a woman suffering from common respiratory infection with pharyngolaryngitis/tonsillitis. Influenza and bronchitis are taken as wrong diagnoses.

Figure S13. Treatments of Case 3

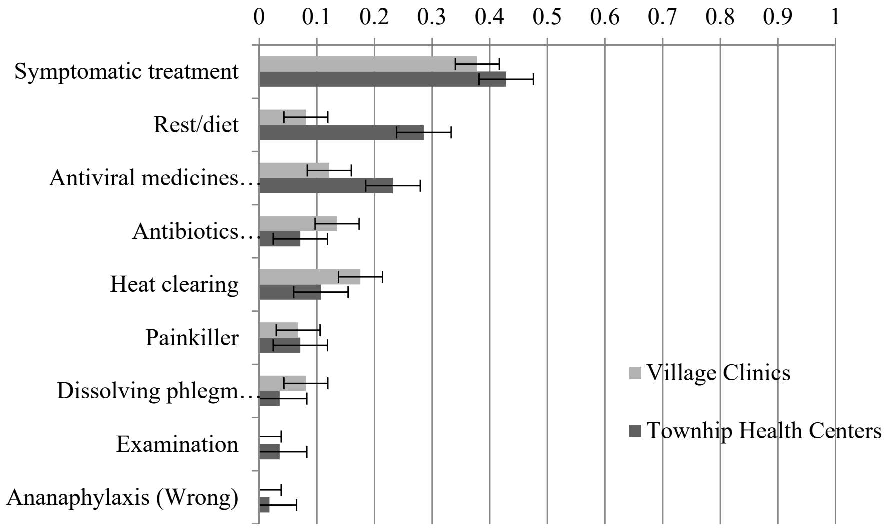

Notes: Considering Case 3 is a woman suffering from common respiratory infection with pharyngolaryngitis/tonsillitis which is not very severe, antibiotics are not recommended. And common respiratory infection does not have specific antiviral medicines, so these medicines are not recommended.

Figure S14. Diagnoses of Case 4

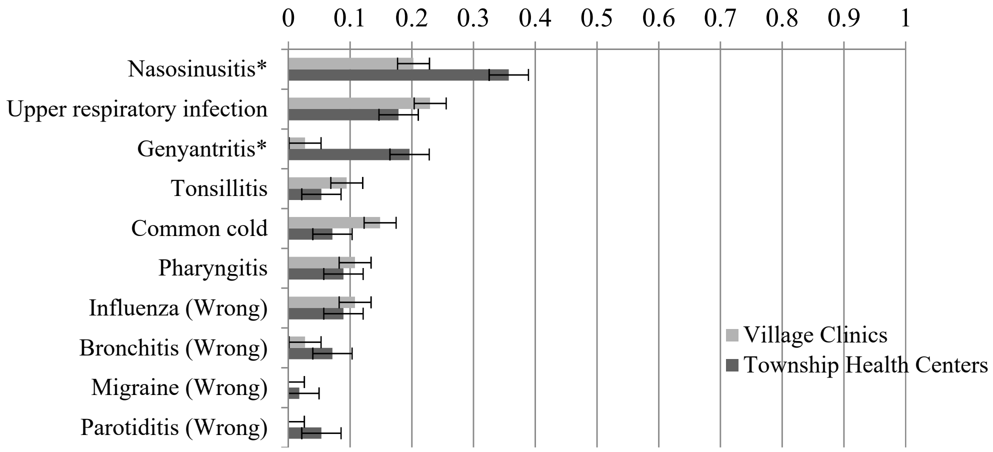

Notes: Case 4 is a man suffering from genyantritis/nasosinusitis. Parotiditis, migraine, bronchitis and influenza are taken as wrong diagnoses. And considering this patient also suffers from tonsillitis/pharyngitis, and some GPs note all these diagnoses, we used bar graph but not pie graph to present the results.

Figure S15. Treatments of Case 4

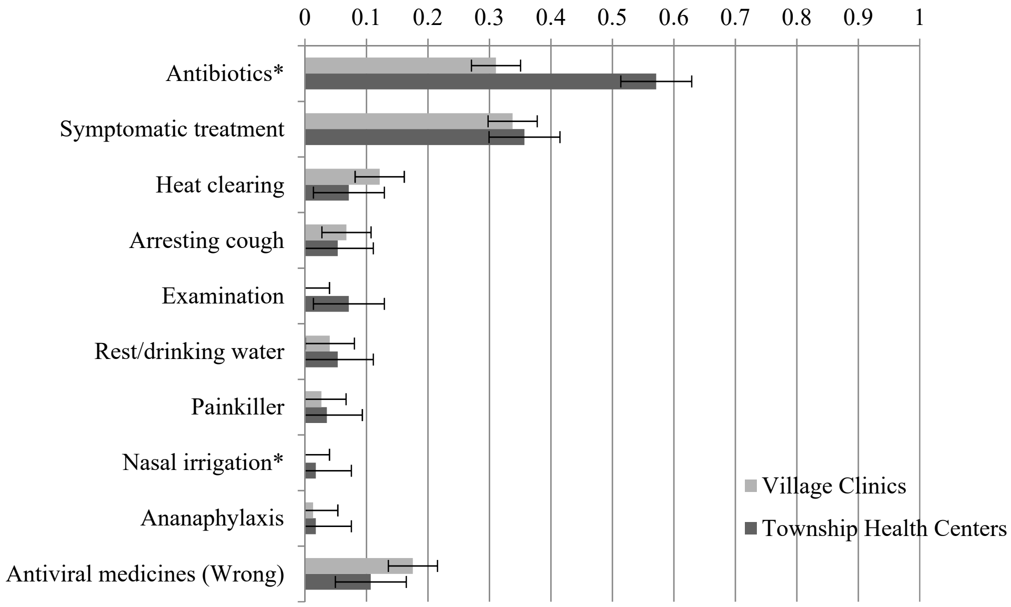

Notes: Considering case 4 is a man suffering from genyantritis/nasosinusitis, antibiotics and nasal irrigation are recommended. As it is not caused by virus, antiviral medicines are taken wrong.
